# One Health, One Microbiome

**DOI:** 10.1186/s40168-025-02231-6

**Published:** 2025-10-23

**Authors:** Abdifatah M. Muhummed, Kayla C. Lanker, Simon Yersin, Jakob Zinsstag, Pascale Vonaesch

**Affiliations:** 1 Department of Fundamental Microbiology, University of Lausanne, Lausanne, Switzerland; 2 Institute of Health Science, Jigjiga University, Jigjiga, Ethiopia; 3 Human and Animal Health Unit, Swiss Tropical and Public Health Institute, Basel, Switzerland; 4 University of Basel, Basel, Switzerland

**Keywords:** One Health, Microbiome, Antimicrobial resistance, Climate change

## Abstract

One Health is a concept and framework for addressing the interconnected nature of humans, animals and their environments to improve the health and wellbeing of all three, along with added social and financial benefits. On a microscopic level, the microbiota is a clear biological connector with strains shared across domains (One Health Microbiome). In this review, we introduce the concept of One Health and the One Health Microbiome and discuss strain-sharing across and within domains. We also highlight its impact on the spread of antimicrobial resistance (AMR) genes as well as overall microbiome diversity and resilience to climate change. Finally, we discuss critical areas for further research and conceptual development, encouraging future research integrating One Health and microbiota-AMR concepts.

## Highlights

- Environmental, animal and human microbiomes are connected, forming together the so-called “One Health Microbiome”.
- Within the One Health Microbiome, there is extensive sharing of strains, contributing to positive outcomes, such as buffering against stressors like climate change, and negative outcomes, such as sharing of pathogens and antimicrobial resistant strains.
- Strain-sharing follows ecological principles, including strain dispersion and environmental/host filtering.
- Strain-sharing is dependent on the taxonomic identity of the strain shared, its capacity to adapt to a new environment and the characteristics of the ecosystem into which it is transferred.
- Microbiomes, especially if they are in a dysbiotic state, are also hubs for the emergence and spread of AMR genes.

## Introduction

Humans and other living organisms as well as ecosystems harbor a vast community of microorganisms, including bacteria, viruses, fungi and (micro)eukaryotes (called the microbiome, Box 1). These microorganisms are crucial to their host’s health as well as in environmental domains such as soils and oceans. In humans, the microbiome is acquired during the birthing period from the mother [1–4], and then matures during the first months and years of life, until it reaches stability towards the age of roughly 5 years [5]. This early life ecological succession is shaped by several factors, including bacteria acquired vertically from the mother, horizontally from other family members as well as from the broader environment, including food, animals, as well as the natural and built environment, through a mechanism called dispersal [6]. Once established within the gastrointestinal tract, environmental filtering leads to further shaping of the microbial community. Several factors of the human host itself, but also of the broader environment, have been shown to act on this filtering, including diet [7], lifestyle factors, such as exercise, antibiotic consumption or overall hygiene, socioeconomic status and the broader social and political context (reviewed in [8]). This dialogue is bilateral, as it has been recently shown that the intestinal microbiota is also implicated in dietary preferences [9] as well as in the motivation to exercise [10], thus clearly showing the mutual interrelatedness of microbial community structure, host behaviors and health.

Traditionally, humans and their microbiomes have been studied separately from the other domains that surround them. However, it is now clear that substantial strain-sharing occurs between different humans, between humans and animals, as well as with the broader environment [11]. Given the links between the different domains, the microbiome thus needs to be viewed as a broader construct, where we as humans form a continuum with the microorganisms inhabiting our body, yet also with the broader environment that influences this microbial community. To better describe these interactions, we previously suggested the term “One Health Microbiome” [12], which is the sum of genes and strains shared in between humans, animals, and the environment.

The interrelatedness of human, animal and environmental health has been acknowledged as early as the nineteenth century by the German veterinarian Rudolf Virchow, who coined the term “zoonosis” for a pathology shared between animals and humans. In the 1960ies, this concept was expanded by Calvin Schwabe in the USA into a concept termed back then “One Medicine” [13] and now termed “One Health” [14]. The concept gained in momentum and finally, in 2004, the Wildlife Conservation Society brought together experts in human and animal health and laid the basis of the Manhattan Principles, which call for an international, interdisciplinary approach to prevent disease [14]. They form the basis of the now widely adopted “One Health” concept which is embraced by the Quadripartite organization coordinating the actions of the World Health Organization (WHO), The Food and Agriculture Organization of the United Nations (FAO), the World Organization of Animal Health (WOAH) and the United Nations Environmental Programme (UNEP) with the One Health High Level Expert Panel (OHHLEP) [15].

Regarding the microbiome, bacterial strains are shared within and across domains, shaping the final microbial consortia through mechanisms described in ecology as “strain dispersal” and “ecological filtering”. The human, animal and plant microbiome thus forms part of a “One Health Microbiome” [12] and microbiome research should clearly start to embrace a One Health approach [16].

**Fig. 1:**
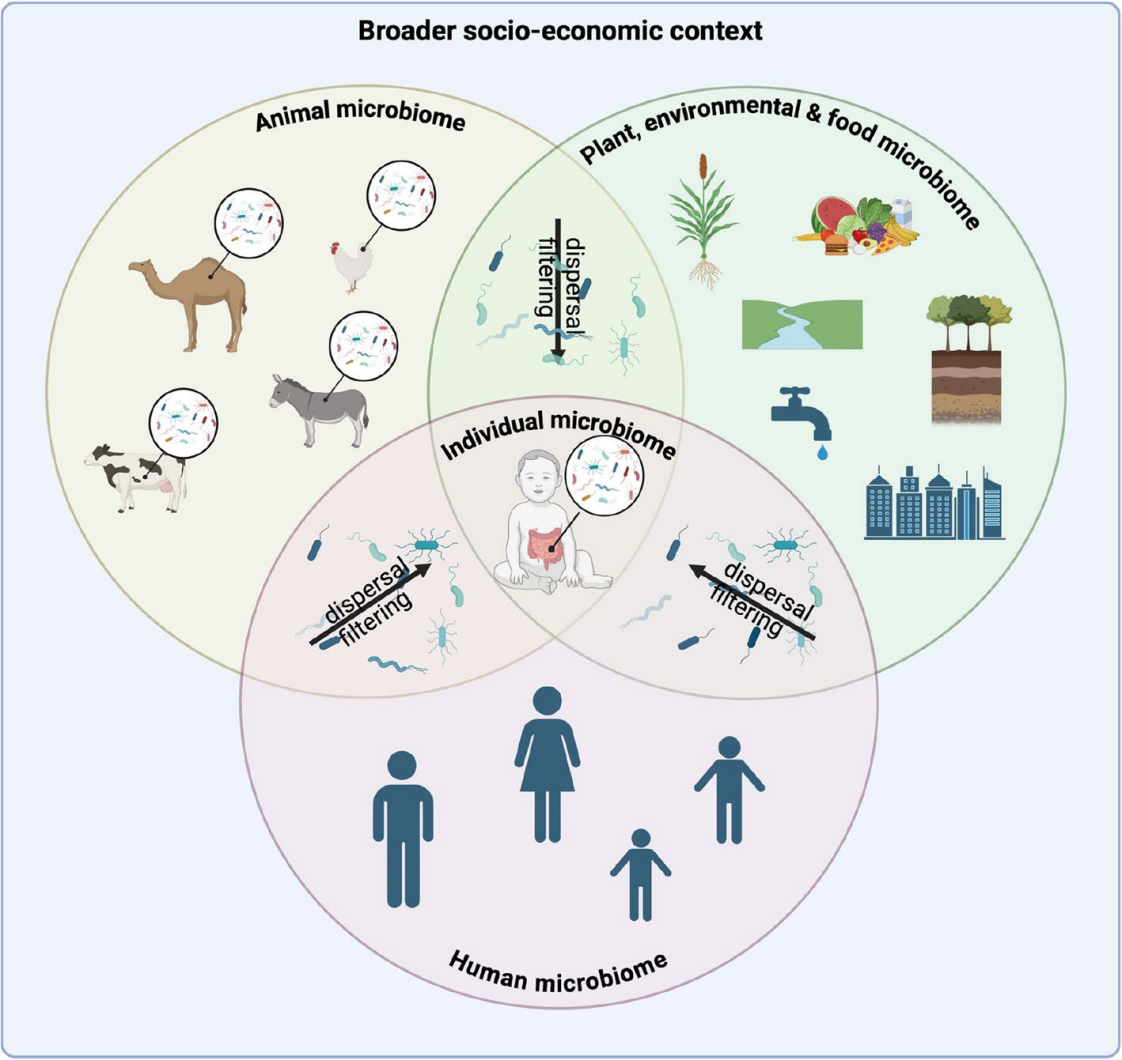
Interconnectedness of the human microbiome with other biomes. There is increasing evidence that microbial strain-sharing does not only apply to a given domain (i.e. human, animal, plant, environmental) but that strains are shared also widely across domains. An individual’s microbiome is thus at the same time a product of different host factors, including the immune system, genetics etc., but also external influences, such as diet as well as strain-sharing from other humans or biomes from unrelated domains. The final microbiome is then shaped by different ecological mechanisms, including dispersal, filtering as well as priority effects. The lifestyles people live across the globe impact the natural strain dispersal, thus leading to a unique microbiome in each individual subject/host

To date, there is only little known about the dispersal and filtering of microorganism within the different, connected ecosystems of this larger framework, but there is no doubt that the different domains are interlinked (Fig. 1). This stresses the importance of ecosystem health to maintain a healthy microbiome and ultimately our own health [17]. Indeed, changes in the microbiomes of other domains, i.e. due to climate change, might have important repercussions on the overall One Health Microbiome, a phenomenon that is still largely unexplored. Furthermore, the loss of interconnectedness with the environment in the urbanized world affects the sharing of strains in between these different compartments, likely contributing to the differences in the fecal microbiome observed across the world. The consequences of this interrupted transmission for our health are only starting to be elucidated.

In this review, we aim to introduce the concept of One Health and of microbiomes in humans, animals, plants and the environment.

We then move on to closely look at the interconnectedness of the microbiome of these different domains, their role in the sharing of potentially harmful bacteria, such as bacteria carrying antimicrobial resistance (AMR) genes, as well as the impact of the loss of exposure to other domains and of climate change on the One Health Microbiome. We finally highlight critical areas for further research and conceptual development, encouraging future research integrating the One Health and microbiota-AMR concepts.

### One Health Microbiome: sharing of microbial strains within the broader ecological network

It has long been known that bacterial strains, especially if they are pathogenic, are not restricted to a single domain, but are shared within a broader ecological framework. Sharing seems to be most prominent at the so-called “ecotones”, zones where different ecosystems intersect, such as the rhizosphere, phyllosphere, mucosal surfaces or the skin, to name a few [18].

To date, most work focuses on the sharing of bacterial strains, mainly using large-scale sequencing datasets. This work has shown that there is extensive sharing of strains within each domain, i.e. between family members [19, 20], mothers and their babies [1–3, 21], including through bacteria present in the breastmilk [22], fathers and their babies [23] or close social contacts [24]. In humans and mice, it has been shown that in the first weeks/months of life, there is at first a dominance of maternally transmitted microbes, with an increasing percentage of socially transmitted microbes as the animals/humans age [24]. Furthermore, inter-individual strain-sharing was found to be both taxon- and host-dependent. On a global scale, *Bifidobacteria* and *Bacteroides* were the most commonly shared bacterial groups, while other taxa were frequently shared in more specific contexts [19, 20]. Additionally, host dependent factors played a role [19], with some individuals serving as “superspreaders”, sharing more strains with others in their community [19]. While the taxon-dependent spreading seems to be linked to mechanisms of survival, including aerotolerance, spore formation or general features leading to better survival outside the host, the characteristics of so-called “superspreaders” are not clear to date.

Strains can also be shared between domains, i.e. between humans and animals, especially if they are close in their physiology and share a common space where bacteria can be transmitted, i.e. through aerosols [25]. These dynamics seem to be more intense the more contact humans and animals have [26] and also includes antimicrobial resistance strains, as shown previously and most frequently in chicken farmers, who share resistant strains with their poultry [26, 27]. Similarly, close interactions between humans and companion animals such as dogs can also facilitate microbial exchange. For instance, a study in Japan reported high gut microbiota diversity in both humans and dogs, yet only 11 out of 5709 ASVs (0.19%) were shared between the hosts [28]. Notably, members of the *Ruminococcus gnavus* group were initially detected in dogs and later found in humans by the third month of cohabitation, suggesting potential host-to-host transfer [28]. Other shared ASVs included *Faecalibacterium*, *Streptococcus* and *Blautia* [28]. Another study reported comparable microbial diversity, with only 5.9% overlap between human and dog dental plaque [29]. Beyond the gut and oral cavity, unweighted UniFrac distances showed that adult dog owners had skin microbiota comparable to their own dogs, with a significant increase in bacterial diversity observed on their hands and foreheads (*p* < 0.001) [30].

Lastly, there is also extensive sharing between the environment and animals, including humans. This can be through food, i.e. the ingestion of fermented food and their bacteria or bacteria present on raw food items [31, 32] and/or through tap water [33] or through close contact with nature, which leads to overall and long-lasting changes in the intestinal and skin microbiome of humans [34]. Likewise, animals and humans can also directly spread strains to the environment, such as through excrement dispersion into soils, water or air.

Thus, there is a clear indication that the sharing of bacteria and other microorganisms, either pathogenic or commensal, is very common in a broader ecological network and that strain-sharing seems to be enhanced in the context of frequent contact and increased bacterial load.

### Strain-sharing within the broader ecological network is taxa specific

Sharing of strains is mostly not stochastic but is governed by specific effects that shape the transmission efficiency as well as the success of colonization in a new environment. The most common model describing sharing of strains is the *dispersion and host filtering* model, where the dynamics of microbiota migration is described through microbiome dispersion, where bacteria can freely move in between different hosts or locations and are then filtered, i.e. through the availability of nutrients, through events of competition, or through restriction by a given host, i.e. the mammalian immune system [35, 36]. Furthermore, previous colonization by closely related bacteria can further restrict colonization of a newly invading strain (“priority effect”). Based on this model, bacteria are divided into generalist and specialist species. Generalists are bacterial taxa that are found in many different environments and can adapt to a wide range of conditions. Specialists are more restricted to a given environment and often adapted to a given task [37]. Overall, specialists seem to be more abundant than generalists across many different ecosystems [37]. Much of the data that is available to date has been gathered through large meta-analyses, pooling data from several different studies, environments and often also timepoints. While broad general principles have been established, the detailed mechanisms governing strain transmission and establishment in new environments remain largely unknown. Of note, many pathogenic bacteria, including species that are often associated with antimicrobial resistance, are generalists. For example, the group of bacteria called ESKAPE (*Enterococcus faecium*, *Staphylococcus aureus*, *Klebsiella pneumoniae*, *Acinetobacter baumannii*, *Pseudomonas aeruginosa*, *Enterobacter* species and *Escherichia coli*), which are a leading cause of nosocomial infections in health care settings worldwide, are all generalist bacteria and facultative aerobes. The ESKAPE bacteria are not only of concern for their ability to easily disperse but can also potentially transfer their resistance genes to other bacteria when they insert into a new environment. Nevertheless, spreading across domains seems, at least in some cases, to be somehow restricted, as no evidence on cross-domain sharing of carbapenem-resistant *Klebsiella* was found in a One Health study assessing for *Klebsiella* transmission in Northern Italy [38]. However, this finding is in sharp contrast to a study in China, where association between the carriage of mcr1-positive *E*. *coli* and aquaculture was found [39]. Furthermore, certain bacterial taxonomic groups frequently found in the human microbiome, such as the Proteobacteria, seem to be enriched in generalists, while others, such as the Bacteroidetes, seem to be enriched in specialists [40]. At lower taxonomic levels, members of the *Mycobacterium* and of the *Streptococcaceae* were more often generalists, while strict anaerobes, such as members of the *Lachnospiraceae*, *Christensenella* and *Prevotella* were more often specialists [40]. Strain-sharing across humans, humans and food as well humans and animals is nicely summarized in a review paper by Heidrich et al. [41], showing extensive sharing of generalists such as *E. coli* and *Lactobacillus* among humans, animals and the environment as well as sub-species speciation in specific domains, i.e. for *Streptococcus gallolyticus* in food vs. human samples. While species are rather highly shared across humans and, to a lesser extent, also animals and environments, strain-sharing is much rarer, with sharing rates decreasing by increasing social distance. The sharing rates in mothers and their 0–3-year-old children are on average ca. 34%, decreasing to 12% in cohabiting adults, and 8% in non-cohabiting twins or adults from the same village not sharing the same household. Across domains, a large meta-analysis showed that up to 3% of all bacteria found in human stool might be of food origin [42]. Similar datasets on companion or livestock animals are currently missing. More work is clearly needed to better understand strain transmission within the One Health Microbiome.

Furthermore, as generalists have been described to more readily diversify and evolve [40], the presence of resistance genes in ESKAPE pathogens and other generalists are of utmost concern.

In conclusion, the main factors shaping dispersion of given taxa is thus the characteristics of the microorganism itself, the environment they try to colonise and finally further filtering steps from the environment.

### Dysbiosis and colonization resistance—a single concept across all microbiomes

The balance between the microbial communities and the biotic or abiotic hosts (termed “eubiosis”) are fragile and are easily disturbed. When disturbed, this can manifest in what is often called a “dysbiotic microbial community” or “dysbiosis”. A dysbiosis can be characterized by a general decrease in microbiota alpha diversity (i.e. the number of different microbial taxa as well as the evenness of their distribution) and/or a decline in protective commensal microbial organisms with a corresponding increase in opportunistic and pathogenic strains. In the intestinal tract of animals and humans, this can further be accompanied by changes in the host physiology, i.e. a thinning of intestinal mucus layer and subsequent invasion of (pathogenic) bacteria into the intervillous space; epithelial damage and blunting of microvilli; and increased inflammation, indicated by an increase in immune cells and inflammatory proteins [43–45]. Long-term, intestinal dysbiosis can lead to widespread chronic inflammation of the gut as well as in distant body sites [45]. Microbiota dysbiosis in humans not only affects the susceptibility to intestinal illnesses but has been linked to many non-communicable diseases [12]. The same effect on disease susceptibility has been found in other animals such as piglets, coral reefs and honeybees [46–48]. Dysbiosis in larger environmental systems have also been studied, such as dysbiosis in plants [49], in marine systems [50], and in agricultural systems [51] and have been linked to a significant decrease in the diversity of agricultural soil and plant microbiota, increasing the vulnerability of plants to illness and soil to climatic events [51, 52].

Beside the breakdown of important functions needed for host-microbe symbiosis, dysbiosis can increase the risk for infectious diseases [53]. This resistance conferred to invading pathogens by the microbiota is termed “colonization resistance”. Colonization resistance is a mechanism describing the exclusion of invading microbes (which might be pathogenic or not) from a pre-existing microbial community. This resistance can be explained over several mechanisms, including the availability of nutrient niches, the presence of directly competing strains or direct bacteria-bacteria interactions leading to the inhibition of invading taxa [43]. Colonization resistance is normally higher in more diverse communities, a mechanism recently termed “nutrient blocking” [54]. Each microbiota has a different set of nutrients available and each microbial organism has different nutrient and ecological niches [55]. Therefore, the *niche overlap* and *competition* between the transferred microbial community into a microbiota will determine the extent to which they colonize and potentially transfer genes to the resident population, including the transfer of antimicrobial resistance genes (ARGs) [56]. Naturally, it is expected that the closer the niche of the transferred microbial organisms to that of the new microbiota is, the easier the merging and colonization will be [57], such as in the case of microbiota merging of pig microbiota in a human pig-farmer’s microbiota [25]. Therefore, the greater the difference in microbial ecosystems, the greater the expected ecological and metabolic hurdle for the incoming microbial organisms and the greater the colonization resistance, although this theory requires further study.

On the other hand, pre-existing niche competitors might also increase colonization resistance. Indeed, it has been previously shown that niche competitors can increase colonization resistance for invading pathogens [58, 59]. In summary, colonization resistance is a complex interplay between direct bacterial interactions and competition for nutrients. While most of these concepts have been described to date in animal microbiomes, the underlying ecological principles very likely expand to all microbiomes across the different domains.

When ecosystems are healthy, we generally expect them to maintain a predominantly eubiotic state. The conferred colonization resistance can however be breached by several means, including antibiotics, drugs and other chemical compounds, as well as host mechanisms such as inflammation. In a recent hallmark paper, it was shown that antibiotics do not only act on pathogens but also extensively on commensal bacteria [60]. Similar mechanisms were also described for other human-targeted and non-human-targeted drugs [61] as well as toxic waste and pesticides in an environmental setting [51, 62]. Lastly, in host-associated microbiomes, inflammation [63] and associated changes in ecosystem oxygenation [64] and exposure to pesticides [65, 66] and other drugs [61] were shown to lead to microbial dysbiosis. All these breaches of colonization resistance can lead to the outgrowth of pathogens, i.e. the outgrowth of plant pathogens in dysbiotic soils [67] or of enteropathogens in the intestinal tract of humans and animals [68]. Thus, while the field of microbiota research is still relatively new, especially in non-human animals and environments, eubiosis is an important ecological indicator of the health of each species and the entire system.

### AMR as a prime One Health Microbiome problem

**Fig. 2:**
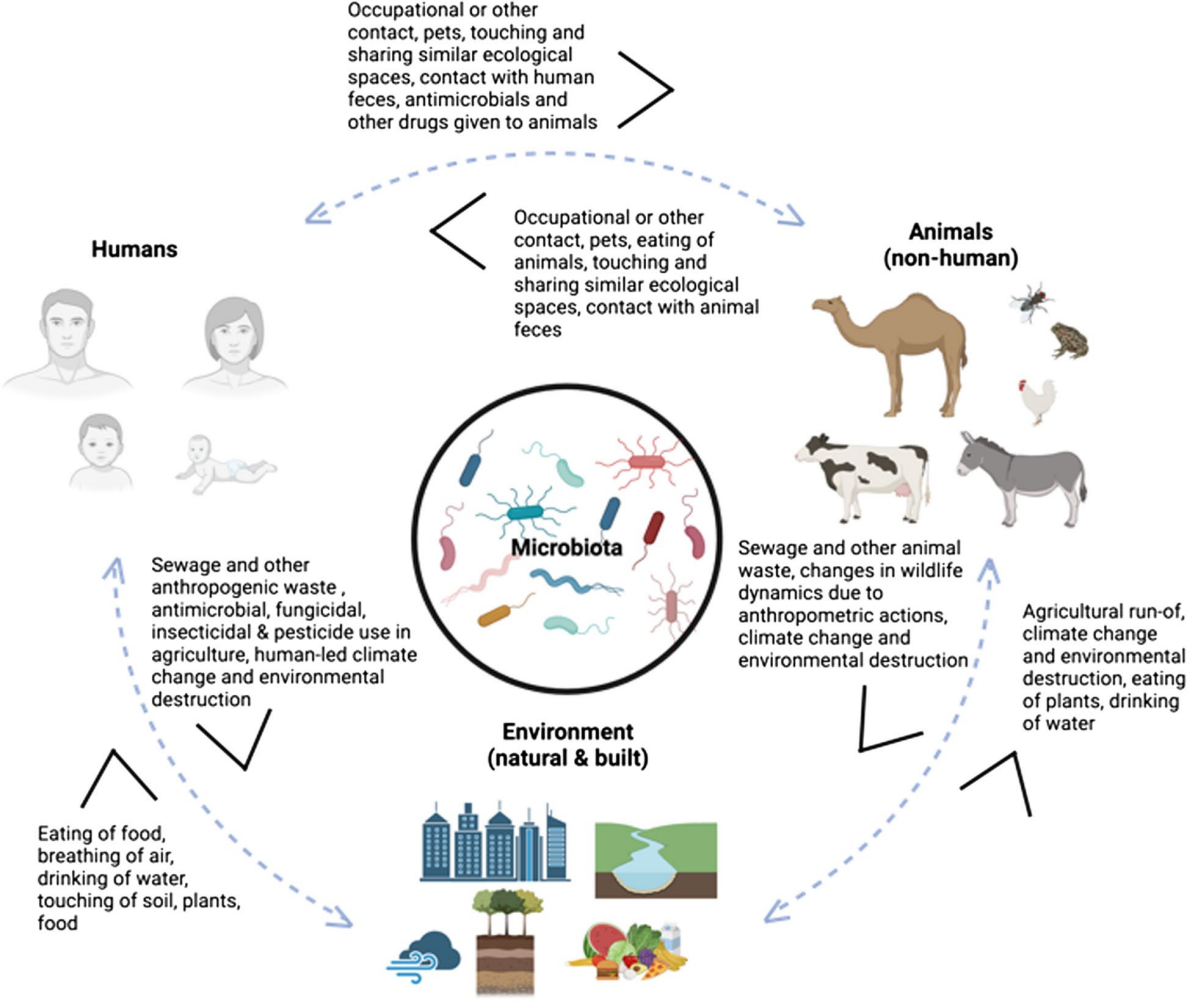
AMR transmission through an interconnected vision of One Health Microbiomes. Increasing evidence points towards addressing AMR within the context of interconnected One Health Microbiomes between humans, animals, plants and environments (natural and built). Direction of sharing of AMR and ARGs has been shown to occur in both directions and via various pathways

An exemplary One Health issue that connects intimately with the microbiota and colonization resistance is antimicrobial resistance (AMR, Fig. 2). AMR is spread through microbial organisms or mobile genetic elements residing in many microbial strains. In humans, AMR bacterial pathogens were associated with an estimated 4.95 million deaths globally in 2019 and 192 million disability adjusted life years (DALYs), an estimate that takes into account those who do not die from illness, but acquire disability and experience a loss of livelihood/income due to illness [69]. It is estimated that by 2050, AMR pathogens will be responsible for over 10 million human deaths per year and cost a cumulative 100 trillion USD globally, surpassing even deaths due to cancer [70]. Severe economic losses due to AMR are also encountered in crop production [71] and livestock rearing with severe impact on food security globally [72]. The recent increase in AMR is largely due to widespread use, sometimes also misuse, of antimicrobial products across agriculture (i.e. seed dressing by fungicides, antifungal and antiparasitic treatments), livestock husbandry (i.e. prophylactic antibiotics) for better growth and human health [73]. The AMR crises in humans and animals is expected to impact low-and-middle-income countries the most, due to intensification of and reliance on livestock farming as a source of income, a lack of adequate sanitation and hygiene infrastructure and a lack of widespread access to newest antimicrobials and health care [74].

Antimicrobial resistance is the adaptive (intrinsic) and/or acquired resistance of a microbial organism to antimicrobial treatment. AMR is evolutionary very old [75], as it is a core weapon in the warfare between different microorganisms. The overall collection of ARGs present in an ecosystem, such as the human intestinal microbiota, can also be termed the “resistome”. AMR genes and pathogens are not restricted to each domain (humans, animals, and the environment) but can be transmitted through microbial and genetic transfers [76] (Fig. 2).

Indeed, as discussed earlier, microorganisms, including resistant ones, can be physically (clonally) transferred through direct contact and through dispersion in air, water and soil. During childbirth, microbial organisms harbouring AMR may also be transferred from mother to newborn, which is concerning since neonates have underdeveloped immune systems and are at risk for infections that can lead to neonatal sepsis and sometimes death [2, 77]. Eating or drinking contaminated animal products such as meat and milk may contribute to transmission, as do poor hygiene and lack of access to sanitation systems, poor disease prevention and access to health care, poor vaccination coverage and a lack of surveillance systems in humans, animals and the environment [78]. Global dissemination of microorganisms and AMR have been demonstrated via human travel and migration [79], animal and other food exports [80], migratory animals [81] and potentially via microplastic biofilms in oceans [82] and long-distance airborne transmission [83].

Besides clonal transmission, AMR can also be shared via horizontal gene transfer (HGT), which is the sharing of AMR genes (ARGs) between distinct microbial organisms. HGT can occur either through transduction [84], conjugation [85], transformation [85] or vesiduction [86]. Conjugation of ARGs is believed to be the most common HGT mechanism between microbiota, as many ARGs are often next to mobile genetic elements on plasmids, which allow for conjugation to occur between different microbial species [85]. Vesiduction, recently discovered, is the transfer of genes through extracellular vesicles (i.e. transport vehicles) between two cells [86], which provide the genetic material more protection from the external environment, such as in biofilms exposed to harsh environments [87]. Thus, microbial populations can rapidly exchange genetic material, including AMR, if a selective pressure is applied. Indeed, HGT, including those encoding AMR, has been demonstrated in and between human microbiomes [88, 89], in and between animal microbiomes [90], in and between plant and environmental microbiomes [91, 92], between plant and human/animal microbiomes [93], between animal and human microbiomes [94] and between animal/human and environmental microbiomes [95]. For instance, a recent multinational study [96] identified 863 distinct microbial resistance genes (ARGs) in gut microbiota, many of which were linked to similar insertion sequences. Among these, 345 ARGs were shared between humans and swine, and 214 ARGs between humans and chickens. One globally widespread resistance gene, *bla*_*-*CTX-M15_ was detected across humans, animals and the environment, often linked to similar sequence types, such as ST131 and ST10, in both low- and middle-income countries (LMICs) and middle-income countries (MICs) [97, 98]. Moreover, *bla*_*-*CTX-M15_ was identified in diverse hosts across high-income countries, with isolate counts of 229 (78%) from human blood stream, 256 (71%) from human faeces, 21 (32%) from sewage, 4 (17%) from slurry and 13 (24%) from cattle [99]. Additionally, despite carbapenems not being authorized for use in animals, *bla*_NDM-5_ was detected in 73.3% of pig farms and 62.2% of chicken, commonly associated with IncX3 plasmids and strongly linked to both human and animal isolates at the plasmid and core genome levels [100].

In addition to food-producing animals, companion animals also warrant attention, as their physical proximity and emotional bonds with humans may create favourable conditions for the spread of microbial and AMR genes. For instance, 102 dog-owning households in Portugal (78) and the UK (24) identified ten households in which *K*. *pneumoniae* (ST556) and *E*. *coli* (ST131, ST2015, ST2179, ST617 and ST963) isolates from both humans and their companion animals carried highly similar resistance genes, differing by no more than seven single nucleotide polymorphisms (SNPs) [101]. Other studies from different countries have provided compelling genomic evidence of antimicrobial-resistant *E*. *coli* and other organisms being shared between humans and their companion animals within households. In Finland, NDM-5-producing ST167 *E*. *coli* was identified in two dogs and one human from the same household, with whole-genome sequencing confirming clonal strains differing by only 1–2 alleles [102]. In New Zealand, faecal testing of 36 pets and 18 cohabiting individuals in 27 households with a human index case of ESBL-/AmpC-producing *E*. *coli* found that 11 households (41%) had at least one other person or pet harboring these resistant bacteria [103]. Clonal *E*. *coli* strains (≤ 10 SNPs difference) were identified in 7 households, including 2 where the same strain was detected in both a human and a pet [103]. Similarly, a study from Australia found that 30% (19/63) of human-animal household pairs carried antimicrobial-resistant organisms, with co-carriage of the same organism in 7 households and whole-genome sequencing confirming clonal transmission in 3 households. Shared organisms included *E*. *coli*, *Klebsiella pneumoniae*, *Enterococcus faecium* and methicillin-resistant *Staphylococcus aureus* (MRSA) [104].

In summary, there is clear evidence that resistance genes and bacterial strains can be shared across domains. The extent and importance of this sharing however remains to be established in larger studies throughout the world, reflecting the different lifestyle and cohabitation schemes of humans, animals and the environment as well as changes in the microbial and resistance profile specific to each geographic site and population.

While it is becoming clear that the microbiome connects all forms of life on earth, there are no studies to date looking systematically at the sharing of commensal microbial strains across different domains. First efforts have started to integrate data of resistant bacteria across several domains, linking mostly strains isolated from water, food or animals with the ones found in humans, occasionally including also the microbiome as a prime hub of resistance sharing (Tables 1 and 2). Jointly, these studies show that AMR is shared mostly within, but also between domains and suggests that future AMR/resistome work should include microbiota analyses, at least on a taxonomic level, to understand the larger ecological context in which the resistome resides. The persistence of AMR (whether bacterial or genetic) in any microbiota can be short term (transient) or long term (embedded) depending on the length of exposure to reservoirs of AMR, as well as other factors such as dysbiosis and antimicrobial use.

**Table 1:** Key studies analysing AMR and the microbiome within a One Health context

Study	Samples	Methods	Key Findings	Limitations/Future directions
Pal et al. 2016 [155]	Metagenomic analysis of publicly available globally distributed microbiota samples (*N* = 864 samples) from various sites on the human body (*N* = 350), various animals (*N* = 145), and various environments (*N* = 369)	Metagenomic analysis	Low taxonomic diversity in humans and animals; High ARG abundance in smog	Weak spatial and temporal relationships; no transmission pathways assessed; represents one of the first studies attempting to assess the resistome across multiple ecological compartments
Luiken et al., 2020 [156]	Localized cross-sectional study of poultry and pig farms in nine European countries: Poultry farms (*N* = 12): dust (*N* = 35), faeces, stool (*N* = 24); Pig farms (*N* = 19): dust (*N* = 44), faeces, stool (*N* = 54)	Metagenomic analysis	High ARG abundance in farm dust and animals; Weak relationship between dust and human stool resistome	Small sample size; differences in resistomes between dust and faeces not fully explored; no longitudinal assessment. Good study on AMR reservoirs and dissemination pathways
Sun et al. 2020 [122]	Longitudinal study on intensive pig farms in China: samples from veterinary students (*N* = 14), farm workers (*N* = 12), pig faeces (*N* = 40), environmental samples (*N* = 55)	16S sequencing, whole genome sequencing, cell culture, phylogenetic tracing, Bayesian network modelling	High microbial and ARG exchange between students and pig farms; 25% ARGs associated with mobile genetic elements (MGEs)	Limited recovery of microbiota post-exposure; requires longer-term studies to confirm dynamic models; HGT role well-demonstrated but needs further validation
Pehrsson et al. 2016 [157]	Longitudinal, spatial study in El Salvador (rural agricultural setting) and Peru (peri-urban industrial setting): faecal (*N* = 226), animals (*N* = 14), environmental (*N* = 185)	16S rRNA sequencing, shotgun metagenomics, whole genome sequencing	Ecological gradient in resistome and microbiome; highest ARGs in humans, animals, latrines; diverse microbiota, lower resistome in soil/water; minimal human microbiota in water; soil had 25–75% human-origin microbiome; Higher AMR protein load in Peru (urban)	Future studies should model their study designs on this study as it is very comprehensive
Alexandre et al. 2023 [158]	Longitudinal study in microbial communities and resistome: chicken stool (*N* = 56), chicken carcass (*N* = 32), soil (*N* = 12), human stool (*N* = 37)	16S rRNA sequencing, shotgun metagenomics analysis, machine learning classification	Differences were spotted in both the microbiome and resistomes across environments and hosts. However, at a finer scale, several clinically relevant ARGs and associated mobile genetic elements were shared between human and broiler chicken samples	The small sample size and low sequencing depth of soil and carcass samples limited their comparability to the other sample types
Mills et al. 2023 [159]	Cross-sectional study on the modifiers of the gut microbiome and resistome in young children from rural Nicarague; children fecal sample (*N* = 57), chicken fecal (*N* = 25), Soil/dust (*N* = 80), water (*N* = 40)	16S rRNA sequencing, shotgun metagenomics analysis	Abundant phyla varied across human, animal and environment, with household soil/dust and contaminated drinking water exerting significant effects on the gut microbiome	A longitudinal study is essential for further understanding the development of the gut microbiome and resistome in early life and its connection to the environment
Want et al. 2020 [160]	Cross-sectional study on metagenomics profiling in China; fecal samples: human (*N* = 119), chicken (*N* = 120), pig (*N* = 120), and soil samples (*N* = 111)	Metagenomics analysis	The soil microbiome contained the highest number of genes, followed by chicken, pig, and human, which had the least. However, in terms of ARGs and genera, humans, chickens, and pigs clustered more closely, while the soil's ARGs and genera differed significantly	Interesting cross-sectional study, including samples from seeral domains

**Table 2:** Key studies investigating antimicrobial resistance (AMR) in the One Health context using molecular techniques

Publication	Domains		Species	Method
Argudín et al. 2015 [161]		Animals, humans, hospital environment	*Staphylococcus epidermidis*	MLST
Yang et al. 2022 [162]		Animals, humans, production environment	No specific targets	qPCR, metagenomic
Hasman et al. 2005 [163]		Animals, humans, meat	ESBL-*Salmonella*	PCR, sequencing
Hong et al. 2019 [164]		Companion animals, humans, veterinary environment	ESC-resistant Enterobacteriaceae	PCR, sequencing
Zhou et al. 2021 [165]		Animals, humans, environment	*Clostridioides difficile*	WGS, MLST
Subramanya et al. 2021 [166]		Animals, humans, environment	*Enterobacteriaceae*	PCR
Bae et al. 2022 [167]		Animals, humans, environment	Food borne pathogens	PFGE
Jaglic et al. 2010 [168]		Humans, milk, environment	*Staphylococcus epidermidis*	PFGE
Yue et al. 2023 [169]		Food, humans, animals	*Enterobacterales*	PCR
Milenkov et al. 2024 [170]		Animals, humans, environment	*Escherichia coli*	WGS
Huber et al. 2010 [171]		Animals, humans, meat and food	*Staphylococcus aureus*	MLST, PCR
Abukhattab et al. 2023 [172]		Animals, humans, environment	*Campylobacter* spp*.* and *Salmonella* spp*.*	WGS
Subbiah et al., 2020 [173]		Human, animal, environment	*E*. *coli*	qPCR, WGS
Aslam, et al. 2022 [174]		Human, animal, and environment	*Klebsiella pneumonia*	PCR
Kasanga et al., 2024 [175]		Human, animal, food and environment	*E*. *coli*	WGS

*MLST* Multi-locus sequence typing, *qPCR* Quantitative polymerase chain reaction, *WGS* Whole genome sequencing, *PFGE* Pulsed-filed gel electrophoresis

Thus, while there are currently few published studies assessing the One Health-Microbiota-AMR triad, there is a clear need for future research in this field.

### Disturbed microbiomes might increase disease transmission as well as AMR emergence and sharing

As discussed above, dysbiosis is a shared feature across all microbiomes and is directly linked with strain invasion. Dysbiotic microbiomes are thus prone to invading pathogens, including strains carrying AMR, and lead to a higher transmission of these strains. This is, in general, due to a disbalance in the overall community structure, often leading to the outgrowth of bacteria that are normally kept at bay, such as facultative anaerobic enteropathogens. Indeed, it has recently been shown that the overall carriage of extended-spectrum beta lactamase (ESBL) bacteria is higher in dysbiotic individuals, i.e. in children suffering from undernutrition [98]. In addition, it is well known that dysbiosis, induced either by under- or overnutrition [105–107], antibiotics [108] or other drugs [109] leads to a colonisation resistance breach and thus provides favourable conditions for pathogen invasion.

Furthermore, dysbiosis is frequently associated with low-grade inflammation, which favours HGT frequency [110] as well as the outgrowth of *Enterobacteriaceae* [63, 111], thus again contributing to the spread of (clinically relevant) AMR strains. Lastly, in newborns, it has been shown that strain transmission from the mother’s faeces at birth is reduced by 50% in the context of maternal antibiotic intake leading to reduced transfer of maternal microbiome to the newborn. This incomplete strain transmission was shown to lead to enhanced transmission of pathogenic strains coming from the hospital setting, again increasing the likelihood of transmission of AMR [112].

In the soil, the frequent use of herbicides and fungicides can lead to toxic metabolites influencing the overall soil microbiome, including plant mutualists such as mycorrhizal fungi or nitrogen-fixing bacteria [113]. Indeed, soils exploited through organic farming had a more diverse microbiome regarding eukaryotes, but a lower prokaryotic diversity [116]. Organic farming was also associated with higher plant growth and decomposition compared to traditional farming [114], suggesting that eukaryotes play a major role in overall soil health. Microbial community changes can also occur through the thawing of permafrost. In this context, it has been shown that there is a higher load of antimicrobial resistance genes in disturbed soil communities, probably due to a shift in overall community composition [115]. Finally, disturbed soils have also been associated with an increase in plant pathogens, leading to higher use of pesticides, and thus leading again to a potential increase in AMR emergence and spread [116].

Thus, dysbiosis, through the breach of colonisation resistance, is a fertile ground for dispersal of pathogens and AMR carrying strains, thus posing a major risk to overall planetary health.

### Broader implications of microbiomes, strain-sharing and interconnectedness in the context of globalization and climate change

**Fig. 3:**
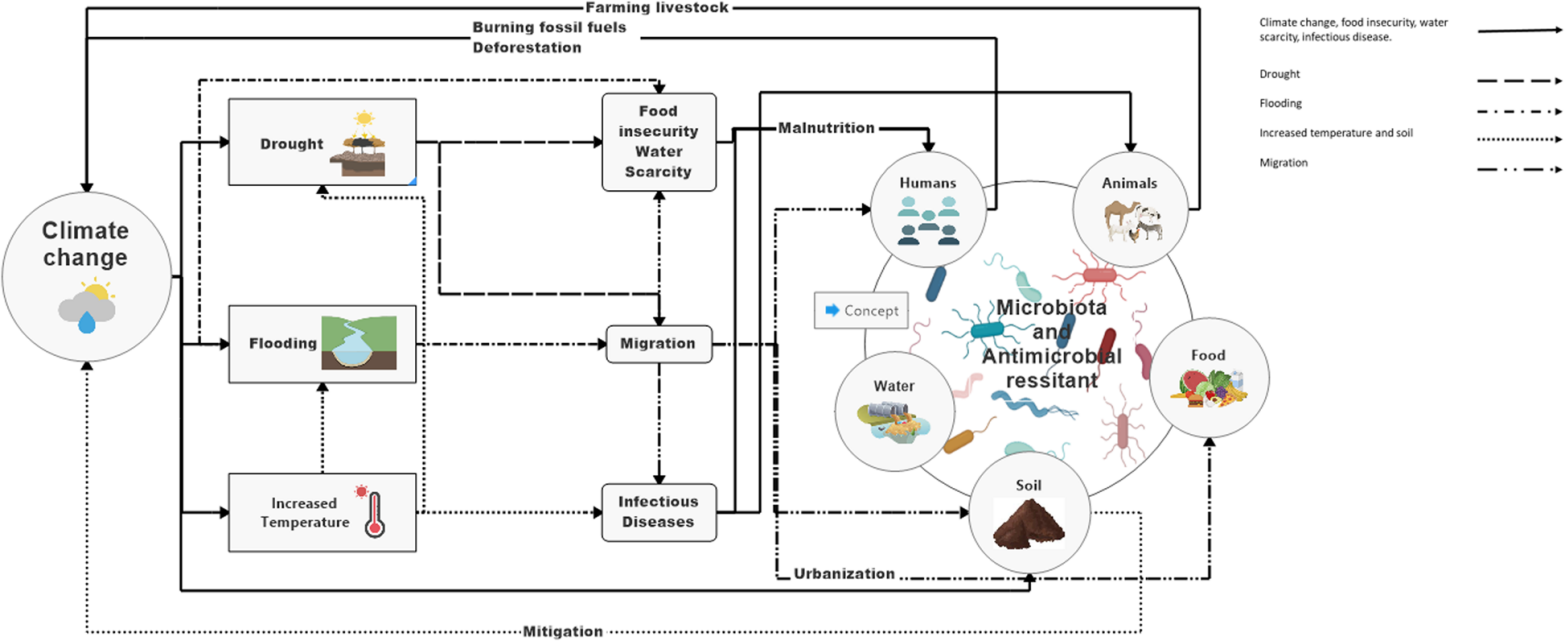
Consequences of climate change and globalization on the One Health Microbiome. Climate change directly affects temperature, drought and flooding, which in turn have consequences including migration, infections and food insecurity. All these factors, either directly or via malnutrition, can affect the environmental, human or animal microbiome, thus having a direct effect on overall diversity and sharing of strains, including AMR bacteria. Inversely, the environmental microbiome can also directly mitigate the impact and further worsening of climate change, i.e. through mitigating the production of greenhouse gases

Microbes are central to climate change and the effects it has on planetary health (Fig. 3), as microbial life is present in everything surrounding us and plays a major role in the global nutrient cycles, including the production and consumption of greenhouse gases and the decomposition rates of organic matter [117]. Global warming has shown a profound, although not yet fully predictable, effect on soils and soil microbiomes [118], and ocean and ocean microbiomes [119, 120], with consequences for nutrient cycling and thus overall planetary health. Furthermore, global warming is likely also increasing the global biological space where microorganisms, animals and the environment interact, as population growth and soil/habitat degradation is leading humans and animals to encroach on new ecosystem spaces, thus leading to new microbial exposures [121]. Finally, weather phenomena such as the El Niño, hurricanes or flooding can lead to changes in ocean and air currents and thus changes in global microbial strain distribution and increased spreading of pathogens, including strains carrying AMR. As an example, after Hurricane Harvey, and the associated flooding, there was a higher density of pathogens in residential areas and agricultural soils [122]. Lastly, water and air permeations during hurricanes were also associated with increased nosocomial infections, thus contributing indirectly to the spread of AMR [123]. The need for studying these interlinked dynamics was deemed so important and timely that a new research field, termed disaster microbiology, was recently founded [124].

Beside direct effects of global warming and severe weather events on bacterial dispersion and community structure, climate change has also led to droughts, desertification and associated food insecurity, war (with associated large fluxes of migration), and an increase in urbanization, especially in low- and middle-income countries. These changes also affect the microbiome, as inter-person spreading of bacteria is increased within urban settings and contact with other biomes from animals and the environment are reduced. Industrialization and urbanization also lead to lifestyle changes, with direct, and likely also detrimental, effects on the human intestinal microbiome, especially regarding overall diversity of the human microbiome, both in humans and animals [125]. There is evidence that this overall decrease in microbiome connectiveness and decrease in human microbial diversity as well as the exposure to air pollution, which has a detrimental effect on the gut microbiome [126], might be linked to the increase in non-communicable disease and to a decrease in overall colonisation resistance to infectious diseases [127]. Similar mechanisms are likely also happening in non-human animals [128], especially in intensively farmed livestock, which are exposed to decreased food diversity coupled with being fed massive amounts of antibiotics and other drugs [128].

Drought also has a direct impact on the composition of the soil [129] and plant microbiome [130] leading to decreased diversity. The concomitant increase in other stressors of the microbiome, such as high salinity or altered pH and increased vulnerability to invading pathogens can further increase the spread of AMR [131]. Furthermore, responses to different stressors, i.e. heat and antibiotics, often lead to similar bacterial adaptations. Indeed, a study on *E*. *coli* showed that exposure to a concomitant stress from antibiotics and a rise in temperature leads to the emergence of AMR co-opted from mechanisms for coping with temperature stresses [132]. Similar effects have been observed in human intestinal commensals where AMR correlated with resistance to other common drugs [61, 133]. This general resistance mechanism seems to be widespread, as shown in a meta-analysis which demonstrated that cell survival in the context of a stressor is increased about tenfold by previous exposure to any other stressful condition, i.e. a temperature rise [134], and likely also through general adaptations, such as increased biofilm formation [135].

Microbiomes, however, can also help mitigate some of the impacts these factors have on their hosts and the broader global ecosystem [136]. Indeed, it has been shown that given bacterial species and overall eubiotic community structure are able to confer crops with increased drought-resistance [137], protect coral reefs against ocean-warming induced bleaching [138] or protect plants [139], humans and animals against invading pathogens [54, 140].

In conclusion, there is now evidence that, some strain-sharing events have detrimental effects, such as the transmission of pathogens, whereas many other strain-sharing events have positive effects on ecosystem functioning or human health, i.e. as a response to increased temperatures, water stress and/or food deprivation. This knowledge could be harnessed to design specific bacterial communities (called also synthetic microbial communities or SynComs) aimed to increase resilience to external stressors in all domains. While efforts in all domains are underway to design such interventions, they largely remain exploratory and are empirical in nature, as we lack a clear understanding of community structure and coalescence, which would aid in predicting intervention success [58]. Nevertheless, the benefit of such interventions, mediating better resilience to climate change, restoring dysbiosed communities and/or conferring means to bioremediate polluted environments will be of utmost benefit to planetary health. However, microbiota-targeting approaches remain challenging to implement as well as difficult to assess in a quantitative manner regarding their effectiveness. Furthermore, as many of the climate change and globalization effects happen on a very broad scale, microbiota-targeting interventions might only be a drop on a hot stone. Practices such as climate control, protecting whole ecosystems, eating a varied diet, using herbal medicines and supporting community-based health approaches and relying on traditional, sustainable agricultural methods may help foster a healthy and diverse environment— and thus the One Health Microbiome. As an example from human health, children that grow up on farms [120] or who are regularly exposed to forest soil [121] tend to have a more diverse microbiome than those with less contact with nature, with positive effects on overall health [121, 122]. Thus, strengthening connectedness within the One Health Microbiome, alongside serious efforts to protect planetary health may be key to reversing the current trend in biodiversity and health loss across all domains.

Undoubtedly, further research is needed to shed light on the complex interplay between microbiomes, climate change and AMR.

## General conclusions

Although the importance of the One Health Microbiome seems to be clear, there are still many questions remaining regarding the nature of and the interactions within the One Health Microbiome, AMR and climate change (Box 2). Future research should rely on interdisciplinary research focusing on the question of how microbes, microbial activity and the associated metabolic fluxes are able to buffer against and are at the same time altered by the climate and especially the rapid and severe changes in precipitation and temperatures that we observe on a global scale. This knowledge will not only be essential to better understand the implication of the terrestrial, urban and aquatic microbiome on the global elemental cycles and how they are affected by climate change, but also to better understand how these phenomena contribute to the spread of pathogens and AMR resistant strains.

Another challenge of the (near) future will be to engineer microbiomes across all domains to confer them a certain resistance to AMR and to climate change-induced community disturbances, allowing for community function to be upheld, as well as the engagement in healthy, meaningful symbiosis and interactions between microbiome and host [58]. For example, protists as members of the cow rumen microbiome directly affect feeding efficiency and the production of methane [141, 142].

Beyond understanding the effect of climate change on microbiomes and trying to buffer its impact through diverse and resilient communities, we thus might also be able to steer microbiomes to release less greenhouse gases in the future, potentially slowing the current climate crisis. Microbial engineering, already a well-established practice in agriculture for crops like wheat, maize, rice, tomatoes, potatoes and peas [143–149], offers promising potential for improving N₂O mitigation. This can be achieved through denitrifiers harboring N₂O reductase [150, 151], utilizing quorum sensing inhibitors to modulate nitrifier activity [152] and employing in situ genome engineering via mobile genetic elements [153]. These approaches provide novel avenues for precise, system-level manipulation of N-cycling microbiomes, leveraging multi-omics technologies and synthetic biology to enhance N₂O reduction pathways while maintaining ecosystem resilience. However, successful implementation requires overcoming technical barriers related to microbial colonization, ecological fitness and regulatory oversight. Integrating these tools into precision agriculture frameworks holds promise for developing robust, scalable and environmentally sustainable N₂O mitigation strategies. Nonetheless, further research is required to fully understand how such biotechnological engineering of microbiomes may affect broader ecological networks and the global ecosystem at large [154]. Finally, it is important to consider that while these mitigation strategies target only localized systems, climate change affects microbe-host interactions on a large scale. Thus, although these discoveries and applications carry significant academic merit, their actual translatability and impact on global warming still needs to be further evaluated.

In conclusion, using a One Health approach to study the One Health Microbiome, AMR and climate change in an integrated manner will not only facilitate the transfer of general concepts across domains, accelerating translational efforts to develop microbiota-targeted interventions, hopefully also reducing associated research costs. Moreover, it will provide valuable insights into the most probable transmission patterns and routes of bacterial strains and resistance genes, thereby enabling more efficient interventions in the evolution and spread of AMR. Incorporating these important variables into models and predictions would allow for the rational engineering of the One Health Microbiome in ways that benefit human, animal and environmental health. Finally, the One Health Microbiome opens philosophical ontological questions on humans, animals and plants as symbiotic interconnected beings. This has implications in anthropocentric academic disciplines like anthropology, sociology, psychology and philosophy of sciences.

### Text Box 1: microbiomes of human, animal and environmental origin

Microbial ecosystems can be found on and inside most complex lifeforms including humans, animals, insects, plants, trees and in water, soil and air [57, 176–178]. In humans and most animals, every opening to the outside environment—our guts, lungs, mouths, ears and skin, etc.—host unique microbiota that are integral to maintaining our health. Animals related to humans such as pigs, dogs or mice have a very similar physiology and functionality of the gut compared to humans, while less related animals like mosquitos or flies have altered morphology, yet still have similar factors influencing microbiota composition [179]. Other living beings like plants likewise have their own set of microbial ecosystems; microbial ecosystems can be found on the leaf surface (phyllosphere), the root system (rhizosphere), and living between plant cells, called endophytes [178]. Functionally, however, the host-microbe relationships in all living beings have similar effects—providing each respective micro-environment with the ability to adapt to environmental stressors (pathogens, heat, toxins, etc.), help in the degradation and extraction of nutrients, influence the immune system of the host and contribute to homeostasis (balance) for all organisms involved [57, 176, 179, 180]. Beside these host-associated microbiomes, the world surrounding us is also filled with non-host associated microbiomes. Indeed, soils [181], water bodies [182] and even the air we breathe [183] harbour distinct microbial communities, that contribute to overall planetary health. The seeding, development and maintenance of the microbiota in various hosts/contexts depends on both environmental and host-related factors. Whether and how microbial organisms will successfully colonize a new environment depends on factors like colonization resistance from the host immune system and resident microbiota, as well as nutrient competition, which is discussed in more detail in this review. The microbiota in plants and the environment are impacted largely by other environmental/climatic factors such as air and water movement (floods, droughts, weather systems), temperature fluctuations (freezing, heat waves), pH, geological features (arid, mountainous, aquatic, etc.), available nutrients in the soil/water/surface and interactions with other living beings [184] and has a crucial role in overall plant health [178]. Thus, while the microbiota of humans, animals and environments may differ in composition, the relationship between the host and the microbiota is similar in all three settings and can greatly impact the health of the host and overall planetary health. There is thus increased interest in engineering these microbiomes to improve host and planetary health. Indeed, successful interventions are described in all ecosystems, including the human microbiome, corals, plants and soils.

### Text Box 2: open questions regarding the One Health Microbiome, AMR and climate change

- What are the rules governing strain dispersion within and between domains in the One Health Microbiome? How is strain-sharing affected by climate change and what are the consequences for planetary health?
- How do bacteria and other microorganisms adapt to stressors in natural contexts, i.e. in full ecosystems?
- What are the ecological factors mediating and predicting clonal transmission and successful colonization in the One Health context?
- What are the seeding sources of each microbiome and how can we restore disturbed microbiomes to allow for better health?
- Which bacteria impede or confer resistance of crops and livestock to heat stress, thus allowing for better crop yields, better food safety, and ultimately better human health?
- Which microorganisms can protect aquatic environments from climate change, thus allowing for functioning nutrient cycles, food safety, and planetary health?
- Can we deduce general rules to engineer microbiomes to protect given environments from climate threats?
- What are the potential consequences for human, animal and environmental health if the interconnected domains that form the One Health Microbiome become disconnected? How is this linked with the current increase in urbanization and improved sanitation?
- How are AMR strains and AMR genes circulated within the One Health Microbiome? Where are hot spots of AMR emergence and transfer? Can they be targeted with effective interventions to halt the spread of AMR, especially for human-relevant microorganisms?
- How are key bacteria, such as the ESKAPE bacteria, shared within the One Health Microbiome? How can a One Health approach be used to decrease the costs needed to control and manage AMR on a global scale?
- What is the role of non-bacterial members of the microbial communities within the One Health Microbiome, the emergence and spread of AMR, the response to climate change and overall planetary health?

## Data Availability

No datasets were generated or analysed during the current study.
